# Morality through the lens of Confucian heritage countries: collective self variations and moral worldviews

**DOI:** 10.3389/fpsyg.2024.1454425

**Published:** 2024-10-02

**Authors:** Reina Takamatsu, Joonha Park, Akiko Matsuo

**Affiliations:** ^1^Faculty of Policy Studies, Aichi Gakuin University, Nisshin, Japan; ^2^Graduate School of Education, Kyoto University, Kyoto, Japan; ^3^Research Center for Advanced Science and Technology, the University of Tokyo, Tokyo, Japan

**Keywords:** morality, self, Confucianism, perspective from non-WEIRD psychology, East Asia

## Abstract

The issue of Western, Educated, Industrialized, Rich, and Democratic (WEIRD) samples dominating research has been ongoing for decades, and now the emerging trend is to turn to theoretical perspectives from the Majority World. Adopting Western-centric methods based on reductionism can overlook important details and differences between similar cultures, particularly in East Asian cultures, where the Confucian values of relational harmony take many forms. We discuss a novel theoretical perspective on moral constitutions in Confucian heritage cultures. Our central tenet is that divergent moral concepts and ideals are present in Confucian cultures because each culture emphasizes a specific pillar and the self is situated differently in the social relationships that define the scope of interpersonal moral obligations. We consider three Confucian countries: China, Japan, and South Korea as examples. Despite geographical proximity and conventional categorization in cultural psychology, each Confucian country manifests distinct patterns of the self, moral ideals, and behavior in socio-moral contexts. To understand how and why moral worldviews vary within a region, we need to examine how the self in socio-cultural contexts differs and guides interpersonal norms and behaviors across sociocultural contexts. We conclude this paper by offering methodological recommendations for including indigenous moral concepts outside the WEIRD context.

## 1 Introduction

The nearly exclusive use of Western, Educated, Industrialized, Rich, and Democratic (WEIRD) samples has been criticized over the last few decades (Arnett, 2008; Henrich et al., 2010). Although many psychologists are aware of this, as evidenced by articles raising this issue having been published in major journals and cited more than 1,000 times, Western hegemony continues to thrive in the psychological field, while non-Western psychology is left behind (Thalmayer et al., 2021). Some methodological approaches focus on identifying universal phenomena (Norenzayan and Heine, 2005); however, non-WEIRD psychology has been missing from mainstream research. Despite the pluralistic stance in dominant theories of moral psychology (Graham et al., 2011; Shweder et al., 1997), Western and non-Western moral psychologists tend to focus on comparing universal moral concepts, reinforcing the prominence of WEIRD psychology while obscuring nuanced cultural differences. This long-standing practice will persist unless novel perspectives are included.

Morality is a sociopsychological topic that has been heavily influenced by Western philosophy. Abstract moral issues and empirical methods have been derived from the moral understanding of researchers in WEIRD cultural contexts (Ellemers et al., 2019; Snarey, 1985). Over the past few decades, a prominent approach in moral psychology has focused on explaining human morality by identifying common patterns and concepts across cultures. While it unifies moral values across borders, one concern is that emphasizing cultural similarities may obscure indigenous views of morality (Obeid et al., 2017). WEIRD people may represent approximately 10–12% of the world’s population, but the rest are simply referred to as a uniform group, such as non-WEIRD or the Majority world. In particular, Eastern perspectives tend to be homogenized in terms of collectivism and interdependence. Moreover, understanding how various moral worldviews prevail within sociocultural contexts is crucial for giving equal weights on the perspectives because intergroup conflicts can arise from perceived dissimilarities in moral ideals (Leach et al., 2007).

As we will see in this paper, East Asian perspectives can provide new insights into variations in the collectivist self and their implications for morality. Western philosophy conceptualizes the self as a separate entity, but morality guides how people interact with others (Rai and Fiske, 2011). Unlike Western emphasis on the self, Eastern emphasis on the interaction between the self and others can explain how moral phenomena are intertwined in the different relational contexts. We posit that, while universal principles exist among all humankind, indigenous standards should be recognized and respected in the study of morality. A critical examination of the sociocultural roots of the people who abide by such standards is essential for understanding morality within a specific context (Rai and Fiske, 2011), and this may only be gained through the lens of the very people who possess implicit knowledge of their norms, values, and ways of life.

### 1.1 Overview

In this study, we draw on indigenous moral concepts derived from Confucianism in China, Japan, and South Korea to examine the convergence and divergence of moral views within a region that shares a common philosophical background. We will first discuss how and why the traditional assumptions in moral psychology that have driven research may not be applicable to non-Western cultures. Next, we use China, Japan, and South Korea, as countries with Confucian heritage, to illustrate how relational contexts shape moral concepts. Studying the influence of Confucianism in East Asia shows how a philosophical doctrine has evolved to prevail as a moral guide for living a virtuous life (Kim, 2016) and at some point, diverged to produce different versions of the moral worldview (Zhang et al., 2005). Lastly, we offer methodological recommendations for studying morality in non-Western contexts. In doing so, we conclude this paper with suggestions for integrating Western and non-Western perspectives to obtain a more complete picture of human morality.

## 2 Part 1: assumptions in moral psychology

The cultural construal of the self provides a framework for explaining cultural variations in motivation, cognition, emotional experiences, and behavior (for review, see Kitayama and Uskul, 2011). However, little attention has been paid to the role of the self in constructing socio-moral experiences. In this section, we present as examples two assumptions that have directed research in moral psychology and explain why these assumptions should consider the self as socially constructed to address moral complexity.

### 2.1 Assumption 1: autonomous thinkers/doers

Western traditions of ethical theory assume that autonomous individuals make moral judgments with the intention of being just (Simpson, 1974). According to Hobbes, moral rules are those that rational, self-interested people would agree to, and those that work to prevent a state of disorganization (Laskar, 2013). In moral judgment tasks, the most common research tool in moral psychology (Ellemers et al., 2019), the researcher assumes that participants make judgments based on their moral sense (Kohlberg, 1984). However, in non-Western cultures, moral judgments may not be private in many instances because the perceived consensus within the larger group may influence individual judgments and behavioral choices (Eom et al., 2016; Vauclair et al., 2015). In collectivistic cultures, the primary regulators of social actions are role expectations within the group and contextual constraints (Hong et al., 1997; Morris and Peng, 1994). Furthermore, members of the community are interdependent, and morally appropriate behavior depends on the context of the relevant interpersonal relationship (Lai, 1995).

Similarly, studies on self-reported traits and moral behavior suggest that a stable moral identity guides moral cognition and behavior. Similar to other social identities, moral identity is considered a core part of an individual’s self-concept and is associated with certain beliefs, attitudes, and behaviors (Aquino and Reed, 2002). Accordingly, Western ethical theories assume that an individual should choose wisely when identifying what virtue outweighs the others in a conflicting situation (Kraut, 2022). In contrast, in non-Western cultures, people may make a moral choice that violates a moral principle with little conflict while still fulfilling interpersonal moral obligations (Miller and Bersoff, 1992). This tolerance for conflicting values in moral choices may be related to the dialectical thinking dominant among people in East Asians and those with East Asian cultural backgrounds. Members of dialectical cultures exhibit more inconsistencies in their self-concept than Euro-Americans (Spencer-Rodgers et al., 2009). Western ethical theories suggest that virtues may conflict in certain situations as individuals strive to stay consistent with their upheld value. In comparison, in non-Western contexts one virtue related to interpersonal responsibilities may override the others (Qingping, 2007). Moreover, people in interdependent cultures have a large set of moral attributes to describe a moral person that can be applied to different contexts, depending on the role and social expectation (Jia et al., 2019), indicating that moral identity is not a stable concept.

### 2.2 Assumption 2: universal moral principles explain the most patterns of moral thinking and behavior in all cultures

Psychological studies of morality have been subject to reductionism in the quest for universal moral principles. Prominent theories, such as the social domain theory and stage theories of moral development, highlight common moral principles (e.g., justice and fairness) across cultures (Kohlberg, 1984; Turiel, 2002) while more recent theories, from a pluralistic view, suggest that moral ideals are contextualized and expressed differently in different places (Atari et al., 2023; Graham et al., 2011; Shweder et al., 1997). Our endeavor to draw attention to moral diversity also resonates with moral pluralist (Cassaniti and Hickman, 2014) and moral relationalist perspectives (Mascolo and Fasoli, 2020). Although we are concerned about reductionism in morality studies, this is not to say that the observed universal moral concepts are not relevant in non-Western cultures. Nor does the dominant theory neglect moral diversity. Our concern is that both Western and non-Western moral psychologists tend to focus their attention away from the notion that moral concept evolve from social interactions (Mascolo and Fasoli, 2020) and may include those specific to the context (Cassaniti and Hickman, 2014). Instead, they use the theories as a guide to find common patterns of moral thinking and behavior across cultures, leading to reductionism.

The moral foundation theory, for example, explicitly holds the pluralistic stance, but given the self-report questionnaire designed for measuring a unified set of moral foundations, most studies based on the theory leave culture-specific moral concepts buried without developing a culture-sensitive measure. Some studies have reported that the moral foundations are common cross-culturally; however, the relevance of some moral situations to the underlying moral foundation may vary (Doğruyol et al., 2019; Murayama and Miura, 2019). For example, the factor structure of moral foundations may be different in a non-Christian cultural contexts (Akhtar et al., 2023) or cultures in which personal moral values are absorbed into collectives (Chung et al., 2016; Du, 2019). Furthermore, the current models may not capture the moral worldviews of ethnic minorities (Davis et al., 2016). These studies suggest that basic moral ideals may be conceptualized differently across cultures; thus, the assumption that universal moral principles should suffice for human morality may oversimplify the experiences and moral concepts of people outside a Western context.

## 3 Part 2: Confucianism

Confucius (551–479 B.C.) sought to restore social order and harmony by reviving traditions of ancestor worship in China, filial piety, and a harmonious feudal society with hierarchical ranks, viewing himself as a successor to antiquity (Nakamura, 1991). Confucianism quickly attracted those in high positions who sought to stabilize their sovereign power. This later became recognized as a school of philosophy that spread throughout China as a powerful force for shaping mentalities and guiding ethical behavior. Confucianism was then imported to Korea and then to Japan around the fourth or fifth century. Although Confucianism was never formally recognized as a religion, Confucian moral ideals have influenced much of the moral education and social life in those heritage countries (Wang, 2004).

Although each pillar of Confucianism is weighted differently, the Chinese, Japanese, and South Korean mentalities are deeply rooted in Confucianism. Song, Yuan and Ming dynasties in China, Tokugawa Shogunate in Japan, and Yi dynasty in Korea adopted Confucianism for hundreds of years. Long before the formal education system was modernized by Western influence, textbooks in the three countries were imbued with Confucian teachings (Stowell, 2003). The common themes revolved around social relations. In contrast to the Western approaches to morality that inflate selfhood, personal cultivation in Confucianism means blurring the boundary between the self and others and cultivating social relationships (Sigurðsson, 2018). Today, Confucian ideals of filial piety, harmony, benevolence, and social hierarchy still exert a strong moral influence (Zhang et al., 2005).

In the era of modernization, each heritage country has undergone economic and socio-political reforms, and in recent decades, East Asian individualism–collectivism orientations have shifted dramatically. More people in East Asia are adopting individualistic values as evidenced by their lifestyles (increased divorce rates, rural–urban migration, more freedom in personal choice; Hamamura, 2012; Steele and Lynch, 2013). However, increased autonomy, life choices, and residential mobility do not necessarily indicate that individualism has conquered collectivism. Similarly, Confucianism has been central to moral worldviews. In Japan, for example, no single religion has bound the people for centuries, but a constellation of Confucianism, Shinto, and the Zen sect make up the Japanese mentality, as seen in Bushido: The Soul of Japan.

### 3.1 Divergence in moral thinking and ideals in Confucian heritage countries

Today, the Confucian ways of thinking continue to flourish in the heritage countries (Hofstede and Bond, 1988), but influence moral cognition and behavior differently, depending on which Confucian value orientation is emphasized in a culture (Chen, 2018). We propose that, within East Asian cultures, the self is situated differently in social relationships and that the extent to which people show moral consideration for others depends on the representation of the self in a given context. Social norms that govern self-other relations, emotions, and communication styles differ among East Asian cultures (Park and Han, 2018), as the ways in which people organize their moral worlds may also differ (Wenxue, 2020). In Confucian heritage countries, harmonious relationships based on trust and mutual obligations to protect one another’s welfare are ubiquitous in daily life, and without explicit transmission from one generation to the next, Confucian virtues are shared among lay people (Lai, 1995). However, people in different East Asian regions differ in how they interpret and emphasize Confucian ethical values (for review, see Chen, 2018; Tamai and Lee, 2002; Zhang et al., 2005). We argue that the divergence originates from the way in which the self is situated in the context, specifically in relation to others.

### 3.2 The self and moral concepts in Confucian-heritage countries: China, Japan, and South Korea

#### 3.2.1 China: lesser self to a greater self and filial piety

Despite the New Culture Movement in the 1910s that banned Confucianism, at the center of Chinese socio-moral, life is filial piety (*xiao*), which binds not only the self and family members, but also the self and others in a close circle (Wenxue, 2020; Yue and Ng, 1999). According to Confucian teachings, people who practice *ren* (benevolence) must love all human beings, and this practice should begin with their parents (Li, 1997, 2008). Originally, filial obligation defined a narrow scope of moral responsibilities, such as caring for one’s parents, showing respect to the elderly, and endorsing the decisions and actions of one’s elders (for a review see, Yeh and Bedford, 2020). Over time, the scope of filial piety has been expanded from parents to others with varying degrees of intimacy, such as friends, neighbors, and colleagues.

The Chinese self-identity is metaphorically viewed as the subordination of a lesser self (*xiao wo*) to a greater self (*da wo*), indicating that the individual (lesser self) encompasses the motivations, interests, and behavioral patterns of significant others (greater self), particularly in family relationships (Barbalet, 2014). This relationship between the self and others is specific to the Chinese culture. The Chinese collective mentality is centered on the Confucian ethic of filial piety (*xiao*), whereas Japanese collective behavior is consistent with loyalty (*zhong*) to group members, another Confucian pillar (Fukuyama, 1995). In close relationships, Chinese students treat friends as family members and are less concerned about being polite and causing trouble than Taiwanese and Japanese students (Uehara et al., 2011).

Confucian justice is based on filial piety, which prescribes the virtue of respecting parents, elders, and ancestors. For outsiders, Chinese filial piety seems to override the moral principle of “justice.” Rather, the Confucian view of justice is based on rules of social exchange that differ according to the relational status and position (inferior/superior) of the individual with respect to the target person (Hwang, 1999). A study shows that, unlike U.S. students, Chinese college students made harsh judgments about illegal conducts done by strangers, but were forgiving of misconducts by their parents (Study 3, Hwang, 2006). A son should hide his father’s misconduct out of filial love even if it means lying and violating the moral principle of justice (Qingping, 2007). The Confucian concept of *yi* (justice) does not align with Western ethical philosophy. In Confucian ethics, members of the society should support close allies, just as filial piety favors close others. In resource allocation, equality means that one should favor the intimate (Hwang, 1999). Unlike Western notions of justice, which use “all things considered” to indicate that all alternatives for individual rights have been weighed, Confucian ethics addresses the concerns of significant others because the lesser self shares experiences and intentions of significant others.

#### 3.2.2 Japan: public and private self

The Japanese concept of *wa* (interpersonal harmony) is reminiscent of Confucian teachings; however, harmony is maintained not only among friends and family members but also among strangers in public. According to the Japanese tradition of dividing the self into private (*uchi*, in-group) and public (*soto*, out-group), maintaining harmony means different things for in-groups and out-groups (Sugiyama-Libra, 1978). With respect to the private self, not all ingroup members fall into this category. For the most intimate, Japanese expect one another to freely express their feelings, intentions, and desires (Midooka, 1990). Compared to other Confucian countries, Japanese people define a narrow circle of intimate (*uchi*) others to whom they apply filial piety. At this point, some may wonder what is characteristic of Japanese Confucianism. To search for uniquely Japanese moral concepts, one should focus on social contexts in which the interaction partner is an outgroup (*soto*) member or an ingroup (*uchi*) member with low intimacy, which we discuss below.

Japanese feel uncertain about other people’s inner thoughts unless the interaction partner is the most intimate, and in such situations, the public self drives self-restrained behavior for the collective goal of maintaining peace. Public spaces apply to any situation in which less intimate others are present. Japanese parents teach their children to be respectful of others and obedient in public, and causing inconvenience or annoyance to strangers in public is considered morally unacceptable (Honda et al., 2017; Takagi, 2013). Japanese college students consider it important to exercise self-control so as not to cause annoyance or discomfort to others in public, whereas Korean students are more concerned with protecting autonomous rights, which is a universal moral value (Jeong, 2020). In public spaces, concern for maintaining the social order and peace guides self-restrained behavior (Yamamoto, 1990).

This concern for public peace may not be equal to a genuine concern for the welfare of others. In particular, the Japanese culture reflects a narrow definition of intimacy, and most social relationships fall into out-groups of varying levels of closeness (Midooka, 1990). The public self regulates collective behavior to maintain order; however, reaching out to help others is not morally obligatory. Among 142 countries, Japan ranked lowest in helping a stranger (Charities Aid Foundation, 2023). For the Japanese, interaction with *soto* others is ideally kept to a minimum because strangers expect one another not to invade their comfort zone. Consequently, Japanese people feel less obligated to help low-intimacy others as long as the public peace is maintained. That is, keeping peace means not interfering in other people’s problems. This behavioral strategy can turn the individual into a bystander. Without understanding the public self, the Japanese may be perceived as distant, rigid, or even cold (Yamamoto, 1990).

#### 3.2.3 South Korea: the influence of familism and social hierarchy roles

South Korea presents a distinctive case for examining the influence of Confucianism on morality, showcasing both continuity and adaptation. The Korean Peninsular has often been described by the general populace and academic scholars as the “most Confucian part of the world,” as it effectively utilizes ancient traditions for the purpose of national development (Koh, 1996, p. 191; Sleziak, 2013). The deep-rooted appreciation for moral values and the endeavor to foster a moral society permeate the educational system, starting from primary school. Here, ethics lessons emphasize hierarchical human relationships, teaching respect for elders, parents, and teachers, as well as care for younger siblings for harmonious community life (Kim, 2014). The role of Confucian ethics is pivotal in shaping perspectives on interpersonal relationships and the dynamics between individuals and society (Moon, 1995).

Geographically positioned between the Chinese continent and the Japanese islands, Korea exhibits a unique amalgamation of mentalities that highlight both filial piety (*hyo*) and loyalty (*chung*). Korean morality shares similarities with Chinese principles, emphasizing interpersonal commitments and an expanded form of familial bonding (Park and Han, 2018). Similar to findings in Chinese studies (Hwang, 2006), the moral judgments of Korean individuals often depend on their perceived closeness to the target (Hyun et al., 2009). The prevalent concept of we-ness (*woori-seong*) operates within close relationships, characterized by a strong sense of bonding, unconditional friendship, mutual altruism, and exclusive favoritism (Choi, 1998). Additionally, akin to Japanese mentalities, loyalty is a fundamental aspect of we-ness. The concept of *nunchi*, literally translating to “eye-measure,” is a crucial skill in interpersonal contexts, maintaining both bonding and loyalty within the hierarchical structures of groups (Robertson, 2019). Unlike Japan, where a clear distinction exists between public and private spheres, in Korea, this distinction is less pronounced due to extended familism. *Jeong*, the sense of attachment between individuals based on reciprocal obligations and mutual care, can also be activated in public once mutual bonding is established (Choi, 2011).

Although limited, cross-cultural comparisons reveal culture-specific features of Korean familism, which is more readily activated even within non-familial relationships compared to its counterparts in Chile and France (Hur et al., 2016). In these countries, familism is typically confined to kinship relationships (Valdivieso-Mora et al., 2016). Likewise, familism in South East Asians (e.g., the Philippines) tends to emphasize more traditional values than the Korean familism (Choi et al., 2018). Literature further suggests that the extended form of Korean collectivism differs distinctly from the collectivism found in other East Asian countries (Choi, 1993; Han, 2017). In contrast to the Chinese and Japanese constructs of self, the Korean self is more agentic, aiming to achieve interpersonal harmony by integrating individual selves into a cohesive whole in social contexts (Inumiya et al., 2007). Supported by the strong influence of Confucian disciplines that emphasize human relationships within hierarchical frameworks, Korean familism nurtures the collective as a cohesive and integrative family-like entity.

### 3.3 Moral disagreement in Confucian countries

Because morality guides how people evaluate others (van der Lee et al., 2017), the real and perceived moral variations within a geographical region may lead to moral dissent. In everyday contexts, perceived and real differences in moral worldviews can impede intercultural exchange. Qingping (2007) argues that Confucian ethics in China prioritize virtues related to family ties over society. This tends to be similar in Korea where the Confucian ethics has been evolved around the family-oriented viewpoint (Kim, 2020). On the other hand, Japanese Confucian ethics focus on public behavior. Japanese people perceive Chinese tourists as being inconsiderate of others because the importance of maintaining public peace differs between the two cultures. However, Chinese students would likely find the Japanese friendship style reserved, distant, and cold because they extend the principle of filial piety (originating from *ren*) to friends, whom they do not consider separate from family members (Uehara et al., 2011). Research on South Korean foreign residents in Japan report similar impression of Japanese classmates or co-workers to the Chinese (Lee et al., 2016). Unlike in China and South Korea, *ren* did not prevail in the Japanese mentality where loyalty to the group triumphed (Chen, 2018). Loyalty may be expressed through self-restraint behaviors, such as disguising one’s emotions or intentions by conforming to the group (Honda et al., 2017). International students in Japan (63.9% from China in the cited study) would find it incomprehensible to exercise restraint in close relationships until they are sufficiently acculturated and can understand the moral meaning of self-control within the host culture (Takamatsu et al., 2021).

## 4 Part 3: recommendations

Morality can be studied from numerous perspectives (Haidt, 2008; Simpson, 1974), implying that any perspectives can be biased if researchers examine an issue solely through their own moral lens. Adopting reductionist research methods can overlook important differences between seemingly similar cultures, particularly those in East Asian cultures, where individuals are more likely to respond to a single context in different ways (Peng and Nisbett, 1999). To show how and why moral worldviews differ among people who are traditionally grouped in the same moral category, we examined how in Confucian cultures (China, Japan, and South Korea) that value interpersonal harmony, the self is situated differently in context, and the scope and content of interpersonal moral responsibilities differ significantly. The same could be said for the other regions of the world, as those in East Asians are not the only non-Western countries (e.g., Cohen et al., 1996). Thus, we provide several recommendations for future studies that aim to elucidate moral diversity among non-WEIRD populations.

To avoid oversimplifying the psychology of people living in non-WEIRD countries, cross-cultural projects must carefully define cultural groups and outline Western and non-Western perspectives at the outset. A minimum requirement for conducting cross-cultural research is to include, in addition to some Western countries, at least several non-Western and non-industrialized countries that do not share a common philosophical, linguistic, and cultural background (Snarey, 1985) for multiple comparisons (Krys et al., 2024). Our examination of the role of Confucianism in the moral worldviews of East Asian countries suggests that classifying cultural groups is not a simple task. Geographical proximity and philosophical background are not reliable guides for categorizing cultures in morality studies (Du, 2019). To categorize cultures, we must examine the historical and sociocultural backgrounds that construct the moral worldview of the people in that place, assuming from the moral pluralist view, that socialization and unique interpersonal experiences within a culture create moral truth (Shweder, 1984). This implies that defining a moral issue involves a conceptual analysis of the issue from the perspectives of the target cultural groups because a moral principle with the same label may carry nuanced conceptual and functional differences across cultures.

Culturally unique morality would remain buried without culturally adapted conceptualizations and tools that take into account the socio-moral practices that govern the place. The pioneering work of Shweder et al. (1997) on the three ethical moral domains is based on the researchers’ ethnographic knowledge of community and family life in the state of Orissa, India, and in-depth interviews with local residents. Using the three ethical codes, Haidt et al. (1993) found that moral concerns among American adults and children and high SES groups are limited to harm and justice, whereas Brazilians and low SES groups have richer moral concepts. Similarly, Vasquez et al. (2001) found that justice was the most salient moral principle for Americans, while Filipinos used the three ethical codes almost evenly depending on the context. More recent studies have unraveled culture-specific morality in East Asian contexts. Jia and Krettenauer (2019) asked participants to describe a highly moral person and found that compared to Canadians, Chinese participants generated more moral attributes including unique descriptions of being moral in the Chinese culture. Similarly, Matsuo (2023) used the situation sampling method to identify the contexts that constitute people’s shared representations of morality and found that, unlike American descriptions of purity that reflect monotheism, Japanese descriptions of purity include some supernatural power (not necessarily an agent) as a motivating force for the moral values of cleanliness and diligence that are essential to Japanese morality. These studies have methodological implications: In order to discover culturally unique moral experiences, the researcher needs native participants to help formulate the moral constructs from the local perspective.

## 5 Coda

Although never organized as a religion, Confucianism has profoundly influenced political regimes, social relationships, and spirituality in the heritage countries. To delineate an indigenous moral perspective, we present China, Japan, and South Korea as examples of countries where the Confucian pillar of relational harmony has been adapted in different ways. Consequently, people within collectivist cultures vary in the scope and content of their interpersonal moral responsibilities. Understanding different or unfamiliar moral concepts requires effort, as people are typically reluctant to change their existing belief system (Hart et al., 2009). However, this is worth the effort. Without understanding the moral world in which people live, misunderstanding or perceiving others as lacking morality can lead to discrimination (Haslam and Loughnan, 2014). In conclusion, our hope is that the study of indigenous moral psychology will not only lead to the discovery of neglected values in moral psychology, but will also promote understanding among those who are divided.

## Data Availability

The original contributions presented in the study are included in the article/supplementary material, further inquiries can be directed to the corresponding author.
